# Validating the Strategic Deployment of Blackleg Resistance Gene Groups in Commercial Canola Fields on the Canadian Prairies

**DOI:** 10.3389/fpls.2021.669997

**Published:** 2021-06-10

**Authors:** Justine Cornelsen, Zhongwei Zou, Shuanglong Huang, Paula Parks, Ralph Lange, Gary Peng, W. G. Dilantha Fernando

**Affiliations:** ^1^Department of Plant Science, University of Manitoba, Winnipeg, MB, Canada; ^2^Canola Council of Canada, Winnipeg, MB, Canada; ^3^InnoTech Alberta, Vegreville, AB, Canada; ^4^Agriculture and Agri-Food Canada (AAFC) Saskatoon, Saskatoon Research Centre, Saskatoon, SK, United States

**Keywords:** *Leptosphaeria maculans*, canola, blackleg, disease resistance, avirulence alleles, major resistance genes, R gene rotations

## Abstract

Blackleg, caused by the fungal pathogen *Leptosphaeria maculans*, is a serious threat to canola (*Brassica napus* L.) production in western Canada. Crop scouting and extended crop rotation, along with the use of effective genetic resistance, have been key management practices available to mitigate the impact of the disease. In recent years, new pathogen races have reduced the effectiveness of some of the resistant cultivars deployed. Strategic deployment and rotation of major resistance (R) genes in cultivars have been used in France and Australia to help increase the longevity of blackleg resistance. Canada also introduced a grouping system in 2017 to identify blackleg R genes in canola cultivars. The main objective of this study was to examine and validate the concept of *R* gene deployment through monitoring the avirulence (*Avr*) profile of *L. maculans* population and disease levels in commercial canola fields within the Canadian prairies. Blackleg disease incidence and severity was collected from 146 cultivars from 53 sites across Manitoba, Saskatchewan, and Alberta in 2018 and 2019, and the results varied significantly between gene groups, which is likely influenced by the pathogen population. Isolates collected from spring and fall stubble residues were examined for the presence of *Avr* alleles *AvrLm1*, *AvrLm2*, *AvrLm3*, *AvrLm4*, *AvrLm5*, *AvrLm6*, *AvrLm7*, *AvrLm9*, *AvrLm10*, *AvrLm11*, *AvrLepR1*, *AvrLepR2*, *AvrLep3*, and *AvrLmS* using a set of differential host genotypes carrying known resistance genes or PCR-based markers. The Simpson’s evenness index was very low, due to two dominant *L. maculans* races (*AvrLm2-4-5-6-7-10-11* and *AvrLm2-5-6-7-10-11*) representing 49% of the population, but diversity of the population was high from the 35 *L. maculans* races isolated in Manitoba. *AvrLm6* and *AvrLm11* were found in all 254 *L. maculans* isolates collected in Manitoba. Knowledge of the blackleg disease levels in relation to the *R* genes deployed, along with the *L. maculans Avr* profile, helps to measure the effectiveness of genetic resistance.

## Introduction

Blackleg, caused by the fungal pathogen *Leptosphaeria maculans* (Desm.) Ces. & de Not, is an economically important disease of canola (*Brassica napus* L.) in many parts of the world, including western Canada, due to yield loss and trade conflicts (Fitt et al., 2006; Van de Wouw et al., 2016; Zhang and Fernando, 2018). Recommended practices to minimize disease impact consist of an extended crop rotation ensuring at least a 2-year break between canola crops, crop scouting and proper pathogen identification, use of cultivars resistant to blackleg, rotation of blackleg resistance sources, and foliar or seed treatment fungicides (Canola Council of Canada, 2020). In Canada, blackleg resistance ratings are determined prior to cultivar registration using procedures defined by the Western Canada Canola and Rapeseed Recommending Committee (WCCRRC). When blackleg-resistant cultivars were introduced in the late 1990s and early 2000s, incidence levels of the disease dropped well below 5% across the Canadian prairies (Kutcher et al., 2010b). Over the last 10 years, blackleg disease incidence levels have been slowly increasing, raising the question of what is happening to the resistant cultivars being deployed (Hwang et al., 2016). Exploration into stewarding resistant cultivars has become a priority for the Canadian canola industry.

Canada is the largest producer of canola globally. In 2019, there was 8,571,700 ha (21.2 million acres) seeded to canola in Canada, which produced 19.6 million metric tons (Statistics Canada, 2019). Seeded acres of canola have doubled since the early 2000s, bringing changes to the level of blackleg disease. Provincial governments in western Canada periodically conduct blackleg disease surveys for the prevalence (the number of fields infected with the disease), the disease incidence (the number of plants within a field infected with the disease), and the disease severity (severity of plants infected rated on a 0–5 disease severity scale). The prevalence of blackleg disease in Canada is around 70% of fields surveyed, showing evidence of the disease. In 2019, the incidence levels were 10, 11, and 10% for Manitoba, Saskatchewan, and Alberta, respectively (Canadian Plant Disease Survey, 2020). Blackleg disease incidence numbers have also doubled since the early 2000s and have increased due to intensified canola cropping frequencies and a shift in the *L. maculans* race profile (Harker et al., 2015; Zhang and Fernando, 2018). Management of the disease has relied heavily on proper identification of the disease or crop scouting, extending out the canola crop frequency, and the use of resistant cultivars to blackleg.

Canola relies on two types of resistance: major gene resistance (also known as qualitative resistance) and minor gene (quantitative) resistance. The *Leptosphaeria maculans-Brassica napus* pathosystem follows the gene-for-gene interaction model but with some exceptions to the model (Flor, 1971). If the major resistance gene matches the avirulence allele within the *L. maculans* population, the plant will initiate a hypersensitive interaction or incompatible interaction, killing the cells around the infected cell and stop the pathogen from spreading any further (Rimmer, 2006). One exception to the gene-for-gene model is the dual specificity of the single avirulence gene *AvrLm1*, which is recognized by both *Rlm1* and *LepR3* (Larkan et al., 2013). Another exception is when an isolate is characterized to carry *AvrLm4-7* or *AvrLm7*, a “hide-and-seek” interaction occurs, which renders *AvrLm3* and *AvrLm9* ineffective within the isolate (Plissonneau et al., 2016; Ghanbarnia et al., 2018). Shifts in *L. maculans* race profile can render blackleg-resistant cultivars less or ineffective. Across western Canada, two dominant *L. maculans* races were identified in 2010 and 2011 (*AvrLm2–4–6–7* and *AvrLm2–4–6–7–S*) and 55 less common races were detected, indicating that diversity is high (Liban et al., 2016). Regional monitoring over time has revealed changes within the population due to the use of resistance genes in many canola cultivars (Kutcher et al., 2007; Liban et al., 2016; Fernando et al., 2018; Soomro et al., 2020); the avirulence gene *AvrLm3* had become scarce in the *L. maculans* population due to the overuse of *Rlm3* resistance gene in Canadian *B. napus* germplasm. The recent increase of *AvrLm7* and *AvrLm4-7* within the population (Liban et al., 2016; Soomro et al., 2020) has also masked the effect of *AvrLm3* and *AvrLm9* (Plissonneau et al., 2016; Zhang et al., 2016; Ghanbarnia et al., 2018). Similar scenarios have occurred globally. For example, the commercial use of *Rlm1* in France resulted in a decrease of the proportion of isolates carrying *AvrLm1* (Rouxel et al., 2003) and in Australia, “sylvestris” resistance was overcome within 3 years after commercial release (Sprague et al., 2006; Van de Wouw et al., 2010). Kutcher et al. (2010b) suggested that improved understanding of the genetic interactions between *B. napus* and *L. maculans* would help to deploy resistant cultivars in time and space to allow for durable resistance. Further knowledge gained on *B. napus–L. maculans* interactions could help alleviate selection pressure from deploying race-specific resistance genes.

An approach that identifies resistance genes in a canola cultivar has been used to better steward blackleg resistance sources. Success of this type of system has been reported in other canola production regions (Ansan-Melayah et al., 1998; Marcroft et al., 2012). In Australia, an intensive cultivar monitoring trial network is used to help predict which *R* genes may remain successful and which genes might have been overcome by virulent populations (Marcroft et al., 2012). This monitoring approach has been able to predict *R* gene failure fairly successfully, avoiding disasters from blackleg disease for farmers (Sprague et al., 2006; Van de Wouw et al., 2014b). Being able to reuse *R* genes in areas where they were overcome previously has also been part of the success to the cultivar rotation system in Australia; it helps preserve advanced genetics and takes some pressure off for the development of new cultivars with novel sources of resistance (Van de Wouw et al., 2014b). Learning from the Australian experience, the Canadian canola industry developed its own R gene-labeling system in 2017 to support the cultivar resistance deployment by farmers.

The new resistance labeling scheme identifies the specific *R* genes deployed within a cultivar, allowing producers to rotate cultivars based on major resistance gene groups (Zhang and Fernando, 2018; Van de Wouw and Howlett, 2019). Previously, if canola farmers were finding increased levels of blackleg within their *R*-rated cultivar, the recommendation was to rotate to a different cultivar (Kutcher et al., 2011). Now, farmers have the option to pick a cultivar based on the *R* genes. The Canadian system places major R genes into groups based on their interactions with *L. maculans* avirulence genes (Zhang and Fernando, 2018), and canola plant breeders can now label known *R* genes into the groups (Table 1). Each group represents *R* gene(s), while “X” represents an unknown or unidentified *R* gene (Canola Council of Canada, 2020). Cultivars continue to be labeled with the resistant (R) or moderately resistant (MR) rating, which rates cultivars based on blackleg severity in comparison to a susceptible check cultivar. Testing is available to farmers for predominant *L. maculans* races in their field through commercial labs in western Canada, providing the field-level information to facilitate better decision making on effective resistant cultivars.

**TABLE 1:** The Canadian blackleg major resistance gene labeling system that classifies *Brassica napus* cultivars’ major resistance genes by lettered resistance gene groups (RG).

Resistance gene group (RG)	Major resistance genes
A	*Rlm1* or *LepR3*
B	*Rlm2*
C	*Rlm3*
D	*LepR1*
E_1_	*Rlm4*
E_2_	*Rlm7*
F	*Rlm9*
G	*RlmS or LepR2*
J	*Rlm5*
K	*Rlm6*
L	*Rlm8*
N	*Rlm11*
P	*LepR4*
X	Unknown

The main objective of this study was to assess the concept of blackleg major *R* gene-labeled cultivar deployment through monitoring the avirulence profile of *L. maculans* population and disease levels in commercial canola fields within the Canadian prairies. The study also intended to develop empirical data on changes of the *L. maculans* avirulence alleles in response to the development of specific *R* genes in these fields. Knowledge gained may help validate the effectiveness of deploying cultivars carrying specific *R* genes in farmers’ fields to manage blackleg disease in Canada. The use of commercial fields in this study provides insight into how farmers have been influencing the *L. maculans* population on their fields through the deployment of cultivars carrying different *R* genes.

## Materials and Methods

### Comprehensive Field Survey

Fields for this project were selected to survey based on their crop history and blackleg major *R* gene group in the canola cultivar grown. Fields with high frequencies of canola were preferable having canola grown back-to-back or every second year. Crop rotation was not a factor in this study as all fields were chosen based on having canola two years prior to the canola crop surveyed. Only fields growing a cultivar with identified blackleg major *R* genes were selected, and the resistance grouping was based on the guideline established through the Western Canadian Canola/Rapeseed Recommending Committee (Table 1; Canola Council of Canada, 2020). Within the farmers’ fields selected, cultivar trials were established by life science companies to test cultivar performance. These cultivar trials were surveyed for this project to compare the blackleg resistance performance of canola cultivars carrying different R genes within the same field. The use of X in this study meant that the cultivar did not have its major resistance genes identified or labeled. Cultivars were represented by resistance gene group.

In 2018, 10 fields were surveyed from Manitoba, eight from Saskatchewan, and 10 from Alberta for a combined total of 28 locations with 77 cultivar samples. In 2019, 11 fields were surveyed from Manitoba, nine from Saskatchewan, and five from Alberta for a combined total of 25 locations with 69 cultivar samples (Supplementary Table 1). Farmers’ fields surveyed were coded with a provincial designation of MB, SK, or AB to represent Manitoba, Saskatchewan, and Alberta, respectively, and a number from 1 to 25 (example: MB5).

### Data Collection

Once fields were identified to survey, overwintered canola residue was collected from each field in spring, and *L. maculans* isolated to help determine the *Avr* profile within the field. Ten isolates were used to represent the pathogen population within a field. Isolates of *L. biglobosa* were common in the overwintered residues, complicating the efforts of getting enough *L. maculans* isolates. At canola plant growth stage of 60% seed color change or prior to harvest, diseased canola plants were collected for the same purpose, with less interference from *L. biglobosa*, of determining the *Avr* profile. The spring and fall samplings were intended to monitor changes in the *L. maculans* population influenced by different *R* genes deployed in the cultivars.

Just prior to swathing or at growth stage 5.2 (seed in lower pods green) to 5.3 (seeds in lower pods green-brown or green-yellow, molted), 50 plants were pulled from each cultivar (10 plants at five sites along a “w” pattern in the field) to assess the field for blackleg severity. Plants were rated for blackleg severity using a 0–5 disease severity rating scale assessing the proportion of blackened tissue at the cross-section of the crown (base of the plant stem) (West et al., 2001; Supplementary Figure 1). Diseased stems were collected, and the pathogen isolated for analysis of *L. maculans* races in a field. Blackleg disease incidence, the percentage of symptomatic plants, was recorded for each cultivar assessed within the field. Over the 2-year period, the 150 cultivars were assessed across the prairie region of Canada.

### Fungal Isolation

The blackleg-infected stubble pieces from the spring and fall field samples for each cultivar were cut into 2-mm pieces then surface sterilized in a 10% bleach solution for 2 min. Once rinsed in sterile water, the pieces were incubated on V8 juice agar [200 mL V8 juice (Campbells, Toronto, ON), 800 mL distilled water, 15 g Difco Agar Technical (BD Diagnostics Systems, Sparks, MD), 0.75 g calcium carbonate (Fisher Scientific, Fair Lawn, NJ), and 0.1 g streptomycin sulfate salt (Sigma-Aldrich, Saint Louis, MO)] amended with 10 mL of streptomycin sulfate. Two Petri dishes per stubble sample were placed on a light bench under cool white, fluorescent light at 22–24°C for 4–7 days. Samples of 10–20 stems were plated per field sample to try to achieve the goal of 10 isolates per sample. Around 5 days post plating, a single pycnidia was picked from the conidial ooze using a fine wire under a dissecting microscope and plated onto a fresh V8 juice agar plate as a single spore isolate; this was duplicated to ensure isolates were gathered from each stem sample. The pycnidia samples grew for 5–12 days on a light bench under the same conditions as the previous step.

### Preparation of Fungal Inoculum and DNA Samples

Pycnidiospores were harvested by flooding *L. maculans* and *L. biglobosa* cultures on the agar plate with sterile distilled water and scraping with a sterilized metal rod to dislodge spores. Spore suspensions were pipetted into two 50-mL sterile centrifuge tubes for DNA extraction (Fisher Scientific, Pittsburgh, PA). Small sterile filter paper disks were placed into the remaining mixture of hyphae, pycnidia, and spores still on the agar plates to capture spores to use for plant inoculation. The soaked disks were then dried and placed into 50-mL sterile centrifuge tubes then stored in the freezer at −20°C.

### DNA Extraction and *L. maculans/L. biglobosa* Differentiation

The DNA samples extracted from fungal isolates were used to differentiate between *L. maculans* or *L. biglobosa*. A mixture of fungal pycnidia, conidia, and hyphae harvested from 8- to 12-day-old cultures was kept in 1.5-mL micro-centrifuge tubes at −20°C, and DNA was extracted by using a modified procedure developed by Lee and Taylor (1990), and Liban et al. (2016). Samples were mixed with a lysis buffer (CTAB extraction buffer), lysed with mechanical beads at 5,000 rpm for 30 s, incubated at 65°C for 0.5 h, extracted with phenol/chloroform/isoamyl alcohol (25:24:1), and precipitated with 95% ethanol by adding 5 M NaCl. The pellet was washed with 70% ethanol twice. Following the final centrifugation, the DNA pellet was dissolved in 100 μL sterile distilled water. To determine if an isolate was *L. maculans* or *L. biglobosa*, ITS-F (PN3): CCGTTGGTGAACCAGCGGAGGGATC and ITS-R (PN10): TCCGCTTATTGATATGCTTAAG primers were used (Mendes-Pereira et al., 2003). The primer set generates 555–560-bp fragment for *L. maculans* and a 580–588-bp fragment for *L. biglobosa* (Supplementary Figure 2). With a 20-bp band difference between the two species, the agarose gel ran for 1 h under 110 V electrophoresis.

### PCR Genotyping for Avirulence Alleles

Multiplex PCR developed by Cozijnsen and Howlett (2003) was used for mating types and avirulence allele characterization of *L. maculans* isolates. DNA samples from *L. maculans* isolates were used for *AvrLm1*, *AvrLm2*, *AvrLm3*, *AvrLm4-7*, *AvrLm6*, *AvrLmJ1/5/9*, *AvrLm10*, and *AvrLm11* using the appropriate primers (Table 2). For *AvrLm4-7* or *AvrLm7*, allele was identified by tetra primer ARMS-PCR (Zou et al., 2018). A marker and methods described by Liu et al. (2020) was used for *AvrLm5/9* to identify *AvrLm5avrLm9*, *AvrLm5AvrLm9*, and *avrLm5AvrLm9*. All other avirulence genes were identified by the presence or absence of their alleles. The PCR reaction included the following reagents: 100–200 ng DNA, 0.25 μL of each primer (10 pmol/μL), 5 μl PCR buffer, 5 μL dNTPs, and 0.5 μL Taq polymerase, filled with water to a total volume of 50 μL. PCR was performed with the following conditions: 3 min at 95°C and 30 cycles of 30 s at 95°C, 30 s at 50°C, 1.5 min at 72°C, and lastly, 5 min at 72°C for extension. The PCR product was visualized after running in 1.5% agarose gel electrophoresis under the condition specified above (Supplementary Figure 3).

**TABLE 2:** Primer name, sequence, product size, and source of avirulence allele primers used in PCR analysis.

Primer name	Sequence (5′–3′)	Product size (bp)	References
*AvrLm1-F*	CTATTTAGGCTAAGCGTATTCATAAG	1,123	Gout et al., 2006
*AvrLm1-R*	GCGCTGTAGGCTTCATTGTAC		
*AvrLm2-F*	CGTCATCAATGCGTTCGG	258	Ghanbarnia et al., 2015
*AvrLm2-R*	CTGGATCGTTTGCATGGA		
*AvrLm3-F*	GAGAGAACTAGTCTGTTAAATGCCTGCTGT	1,357	Plissonneau et al., 2016
*AvrLm3-R*	GAGAGACTCGAGCGCGCTTATGTTAGAATC		
*AvrLm4-7-F*	TATCGCATACCAAACATTAGGC	1,433	Parlange et al., 2009
*AvrLm4-7-R*	GATGGATCAACCGCTAACAA		
*AvrLmJ1/5/9-F*	ACAACCACTCTTCTTCACAGT	479	Van de Wouw et al., 2014a
*AvrLmJ1/5/9-R*	TGGTTTGGGTAAAGTTGTCCT		
*AvrLm6-F*	TCAATTTGTCTGTTCAAGTTATGGA	774	Fudal et al., 2007
*AvrLm6-R*	CCAGTTTTGAACCGTAGTGGTAGCA		
*AvrLm10A-F*	TCAAAAAGCGGCCTTCTC	669	Petit-Houdenot et al., 2019
*AvrLm10A-R*	GAAGTTAAGAGAGCAGGTGAGG	288	
*AvrLm10B-F*	GCGACAGGAATCACAACCTT		
*AvrLm10B-R*	GCCTACGCCAATCTCCAATA		
*AvrLm11-F*	TGCGTTTCTTGCTTCCTATATTT	359	Balesdent et al., 2013
*AvrLm11-R*	CAAGTTGGATCTTTCTCATTCG		

### Avirulence Phenotyping Through Cotyledon Inoculation Tests in Greenhouse

*Leptosphaeria maculans* isolates were used to inoculate a set of differential *Brassica* lines carrying known *R* genes (Table 3) to observe the phenotypic reaction and identify the corresponding avirulence genes carried in the isolates (Supplementary Figure 4). The presence of avirulence genes in *L. maculans* isolates was determined based on symptoms on cotyledons after inoculating. Inoculum concentration was adjusted to 2 × 10^7^ spores mL^–1^ from the harvested cultures derived from a single pycnidiospores cultured on a V8 medium plate. Differential lines were seeded in Sunshine growth mix and put in a growth chamber at a nighttime temperature of 16°C and a daytime temperature of 21°C, with a 16-h photoperiod (Rashid et al., 2018a). For the inoculations, 10 μL of spore suspension (2 × 10^7^ spores ml^–1^) was deposited on each lobe of 7-day-old seedlings, which were wounded with a modified tweezer. Inoculated pots of cotyledon plants were fertilized on the second day after inoculation. Five and 10 days post inoculation, true leaves were trimmed to delay the cotyledon senescence. Six plants were used for each line–isolate interaction, and each lobe of cotyledon was inoculated (four per plant). Westar was used as a control to test the virulence of isolates as it is a susceptible cultivar to blackleg. Symptoms on cotyledons were scored 14 days post inoculation using a disease rating scale of 0–9 (“0” indicating no infection and “9” indicating a large leaf lesion) based on lesion size, chlorosis or necrosis, and presence of pycnidia (Kutcher et al., 2007). A mean of 6.1–9.0 was considered a susceptible (S) reaction, 4.6 to 6.0 an intermediate (I) reaction, and less than or equal to 4.5 a resistant (R) reaction (Zhang et al., 2016; Supplementary Figure 5). If an isolate was characterized to carry *AvrLm4-7* or *AvrLm7*, phenotyping for *AvrLm3* and *AvrLm9* would not be carried out for the isolate due to the “masking effect” (Plissonneau et al., 2016; Ghanbarnia et al., 2018). If an isolate did not carry *AvrLm4-7* or *AvrLm7*, it was tested for the interaction on another two cultivars carrying *Rlm3* (01-22-2-1) and *Rlm9* (Goéland). Each isolate–host interaction was used to deduce the avirulence allele carried by the isolate.

**TABLE 3:** Canola cultivars with corresponding resistance genotype used as differentials to identify avirulence genotypes of *Leptosphaeria maculans* isolates.

Cultivar	Resistance genotype	References
01-23-2-1	*Rlm7*	Dilmaghani et al., 2009
Surpass 400	*Rlm1, RlmS*	Van de Wouw et al., 2009
1065	*LepR1*	Kutcher et al., unpublished
1135	*LepR2*	Kutcher et al., unpublished
Jet Neuf	*Rlm4*	Gout et al., 2006
Westar	*No R gene*	Balesdent et al., 2002
TopasRlm1	*Rlm1*	AAFC-SK
TopasRlm2	*Rlm2*	AAFC-SK
Forge (*B. juncea*)	*Rlm6*	Rashid et al., 2018b
02-22-2-1	*Rlm3*	Gout et al., 2006
Goéland	*Rlm9*	Balesdent et al., 2006

**Cultivars used from Agriculture and Agri-Food Canada Saskatoon are identified as AAFC-SK.**

### Statistical Analysis

Analysis of variance (ANOVA) was used to compare means as an initial statistical analysis tool using the PROC MIXED procedure of SAS 9.4 (SAS Institute Inc., Cary, NC) (Littell, 2006). Disease incidence (DI) was transformed using an arcsine root square transformation, and a log transformation was performed for disease severity (DS) (Shah and Madden, 2004; Ghanbarnia et al., 2011; Rashid et al., 2020) to improve the normality of data distribution. When ANOVA was significant (*P* < 0.05) for DI and DS among resistance gene groups, the means were separated using the Tukey-Kramer test. The Tukey-Kramer test with a probability level for significance of 0.05 was used due to unequal sample sizes (Day and Quinn, 1989). Resistance gene group, year, and province were considered fixed effects.

Diversity and evenness of the *L. maculans* population were calculated using Simpson’s index of diversity (IOD) and index of evenness (IOE), respectively (Simpson, 1949). The IOD is calculated by weighing the number of *L. maculans* races relative to the total number of *L. maculans* races tested. An index of 1 is considered a random or diverse population, whereas an index of 0 would consist of a single race. The IOE is a measure of the relative abundance of different *L. maculans* races in the population, whereas an index of 1 indicates even representation of all races and an index of 0 indicates unequal representation of races.

## Results

### Disease Incidence and Severity by Major Resistance Gene Group

A total of 146 cultivars over 2 years were surveyed for disease incidence and severity. The mean disease incidence, severity, and severity of infected plants was summarized by cultivar’s resistance gene group (Table 4). The resistance gene groups are based on the Canadian blackleg major resistance gene-labeling system that was introduced in 2017 (Canola Council of Canada, 2020). Four blackleg major resistance genes were commercially available during the study, which resulted in six different resistance gene group combinations from Table 1, namely, AC (*LepR3, Rlm3*), ACG (*LepR3, Rlm3, RlmS*), C (*Rlm3*), CE_1_ (*Rlm3, Rlm4*), CG (*LepR3, RlmS*), and X (unknown or not commercially identified major resistance gene).

**TABLE 4:** Blackleg disease incidence, severity, and severity of only infected plants from field sites in Manitoba, Saskatchewan, and Alberta in 2018 and 2019 based on cultivars’ major resistance gene groups.

Resistance gene group (major resistance gene)	Incidence	Severity^a^	Severity of infected only
AC (*Rlm1/Rlm3*)	0.57	0.96	1.65
ACG (*Rlm1/Rlm3/RlmS*)	0.47	0.79	1.36
C (*Rlm3*)	0.36	0.57	1.36
CE_1_ (*Rlm3/Rlm4*)	0.24	0.33	1.19
CG (*Rlm3/RlmS*)	0.25	0.31	1.17
X (unidentified R gene)	0.43	0.62	1.30

**^*a*^Blackleg disease severity rated on a 0–5 severity rating scale (Western Canada Canola/Rapeseed Recommending Commitee [WCC/RRC], 2009).**

Blackleg disease incidence and severity were both significantly different among resistance gene groups (*P* < 0.05) (Table 4). Interactions between resistance gene groups between the years were found to not have a difference on disease incidence or severity. With no difference between the years in this study, it is an indication that disease pressure was consistent between the two growing seasons.

There were significant differences between cultivars with resistance gene group AC compared to CE_1_ cultivars for disease severity (*P* = 0.0326), and additionally, among cultivars with ACG compared to CE_1_ cultivars (*P* = 0.0459) and between cultivars in CE_1_ compared to cultivars in X (*P* = 0.0306). These differences are from all fields surveyed over the 2 years of this study.

**FIGURE 1:**
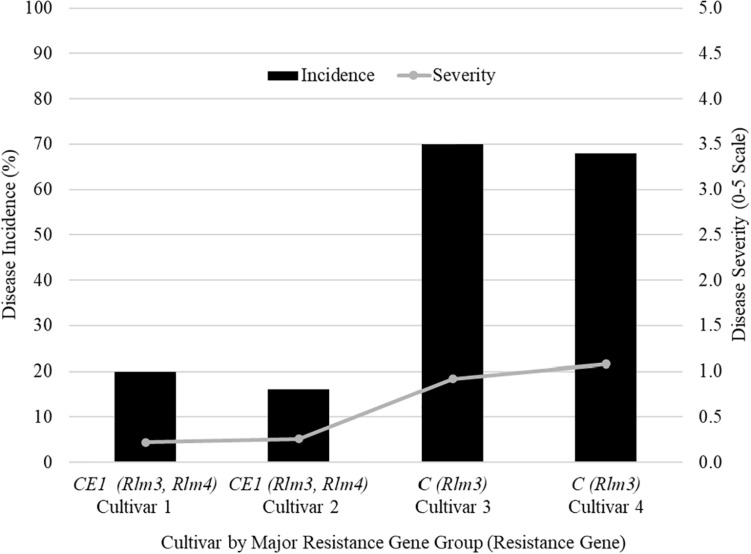
The blackleg disease incidence and severity for field site SK1 in Saskatchewan in 2018 that had four cultivars grown to compare the effectiveness of different major resistance genes.

The farmers’ cultivar trials were where differences can be seen for disease incidence and severity between resistance gene groups. A field in 2018 from Saskatchewan (SK1) shows five cultivars grown in the field identified by their major resistance gene profile (Figure 1). Two cultivars were labeled with C (*Rlm3*), two cultivars with CE_1_ (*Rlm 3*, *Rlm4*), and one cultivar as “X” as its major resistance gene was not labeled for a total of five cultivars (Figure 1). The mean disease incidence was 51% lower in the CE_1_ cultivars compared to the C cultivars. The mean disease severity rating was 0.24 in CE_1_ cultivars and a rating of 1 for C cultivars. The difference between groups is the addition of *Rlm4* in the CE_1_.

**FIGURE 2:**
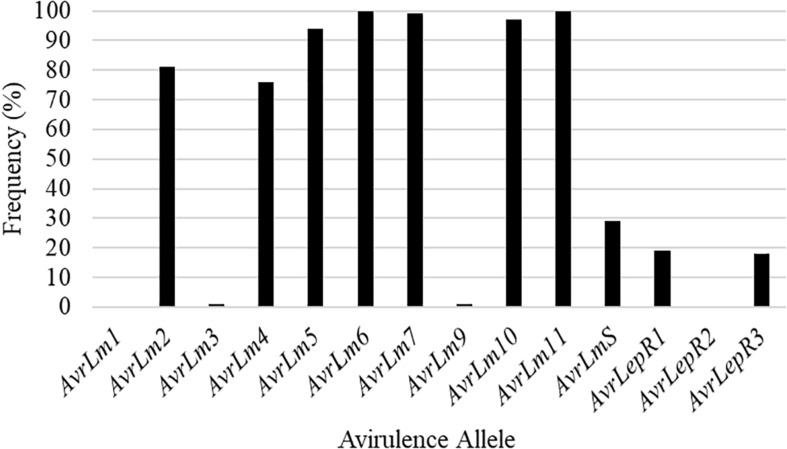
The frequency of avirulence alleles in the *Leptosphaeria maculans* population from 22 isolates collected in the spring of 2018, from a 2016 canola residue, prior to seeding Saskatchewan field SK1 in 2018.

Isolates collected in the spring prior to seeding of the five cultivars identify what the *L. maculans* avirulence profile was in the field. Out of the 22 isolates collected from SK1, 73% had the *L. maculans* race profile of *AvrLm2-4-5-6-7-11* (Figure 2). The remaining 27% was made up of four *L. maculans* races, *AvrLm2-5-6-7-11*, *AvrLm2-5-6-7-11-LepR1*, *AvrLm2-4-5-6-7-11-LepR1*, and *AvrLm2-4-5-6-7-11-(s)* (Figure 2). Figure 2 represents the frequency of *Avr* genes in *L. maculans* population. *AvrLm3* and *AvrLm9* are masked by the presence of *AvrLm7* in the *L. maculans* avirulence gene profile (Plissonneau et al., 2016; Ghanbarnia et al., 2018). This would explain the greater disease incidence in the C resistance gene group cultivars as they rely on the use of major resistance gene *Rlm3*. The frequency of *AvrLm4* in the population was 82% (Figure 2). The use of the major resistance gene *Rlm4* in the two cultivars to *AvrLm4* in the *L. maculans* population inferred the defense response within the plants to initiate disease resistance.

### Deployment of Single-Gene Cultivars vs. Multiple-Gene Cultivars

Single-gene cultivars were compared against all multiple-gene cultivar combinations in this study from all the sites in 2018 and 2019. The cultivars labeled with an X were omitted to complete the analysis as they may have been composed of varying number of genes and combinations. Resistance gene group C was the only single-gene resistance group, while the multiple gene cultivars consisted of four combinations: AC, CG, ACG, and CE1. There was no significant difference between single-gene and multiple-gene cultivars (*P* < 0.05) (Supplementary Table 2). There was also no relationship between years or from the interaction of the multiple gene cultivars and the year.

There is a significant difference in disease severity between cultivars consisting of two resistance genes versus cultivars consisting of three resistance genes (*P* = 0.045; *P* < 0.05). Differences are from the comparison of cultivars in the CE_1_ (*Rlm3*, *Rlm4*) classification and in the ACG (*LepR2*, *Rlm3*, *RlmS*) classification where the differences are found as *AvrLm4* is more frequent in the *L. maculans* population than *AvrLepR2* and *AvrLmS*. The result is higher disease severity in the three gene cultivars as they would only match up to a very small population of *L. maculans* races found across the prairies.

### Frequency of *L. maculans* Avirulence Alleles in Manitoba

**FIGURE 3:**
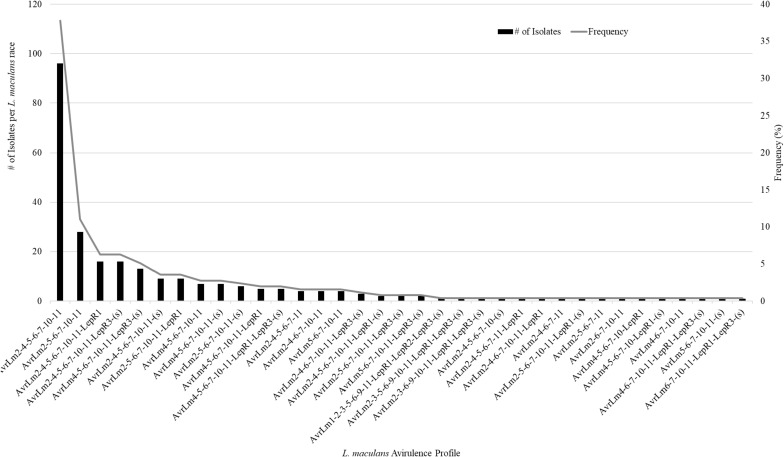
Frequency of 35 *Leptosphaeria maculans* races characterized on the 14 avirulence alleles included in this study. A total of 359 isolates were examined the avirulence alleles, 105 of the isolates were *L. biglobosa.*

**FIGURE 4:**
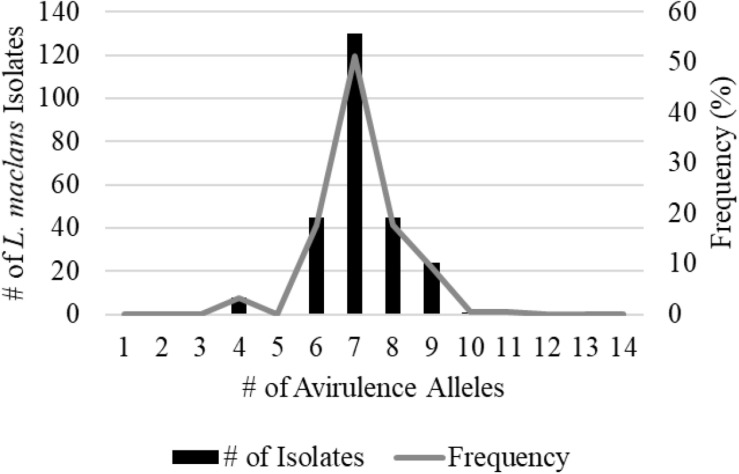
*Leptosphaeria maculans* race complexity based on a total of 254 isolates from Manitoba in 2018–2019 assessed at 14 avirulence alleles.

A total of 359 isolates were characterized for the presence of *AvrLm1*, *Avlm2*, *AvrLm3*, *AvrLm4*, *AvrLm5*, *AvrLm6*, *AvrLm7*, *AvrLm9*, *AvrLepR1*, *AvrLepR2*, *AvrLepR3*, and *RlmS*. Both spring- and fall-collected isolates were used to determine the *L. maculans* races. Figure 3 illustrates the frequency and number of isolates for each *L. maculans* race found in Manitoba. The isolate collection consisted of 105 *L. biglobosa* species. There were 35 unique *L. maculans* races found over the 2-year study in Manitoba. The top two *L. maculans* races only differ by the presence or absence of *AvrLm4*. The most common *L. maculans* race in Manitoba was *AvrLm2-4-5-6-7-10-11* at 38%, followed by *AvrLm2-5-6-7-10-11* at 11% and *AvrLm2-4-5-6-7-10-11-LepR1* at 6%. These three races are the most frequent for both the spring- and fall-collected samples. The next top races from the spring-collected isolates were *AvrLm2-4-5-6-7-10-11-(s)* and *AvrLm4-5-6-7-10-11-(s).* The following top races from the fall-collected isolates were *AvrLm2-4-5-6-7-10-11-LepR3-(s)* and *AvrLm4-5-6-7-10-11-LepR3-(s)*. The *AvrLepR3* gene was only found in fall-collected isolates, and it was not present in any of the spring-collected isolates. The *L. maculans* population evaluated by terms of complexity is the number of avirulence genes carried per isolate. Figure 4 depicts the *L. maculans* race complexity by presenting the frequency for isolates collected in Manitoba by the number of avirulence alleles present. Of the 254 *L. maculans* isolates collected, 51% had seven avirulence genes, 18% had six, and 18% had eight. Avirulence race profiles are shown after the removal of *AvrLm3* and *AvrLm9* due to the “hide-and-seek” interaction with the presence of either *AvrLm4-7* or *AvrLm7* (Plissonneau et al., 2016; Ghanbarnia et al., 2018). The *L. maculans* complexity provides many options to match resistance genes to avirulence genes within the population.

**FIGURE 5:**
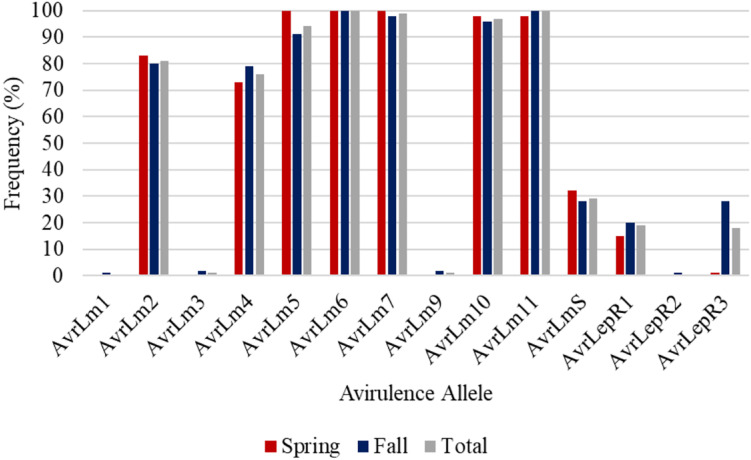
Frequency of avirulence alleles in 254 *L. maculans* isolates collected in Manitoba from 2018 to 2019, assessed for 14 avirulence alleles from spring and fall collections.

*AvrLm6* and *AvrLm11* were found in all 254 *L. maculans* isolates collected in Manitoba in 2018 and 2019 (Figure 5). Of the 92 *L. maculans* isolates collected in the spring, *AvrLm5*, *AvrLm6*, *AvrLm7*, *AvrLm10*, and *AvrLm11* were found in over 98% of the isolates. Similar results were recorded in the fall isolate collection except for lower levels of *AvrLm5*. Three isolates collected in the fall did not have *AvrLm7*, so *AvrLm3* and *AvrLm9* were unmasked. Low frequencies of *AvrLm1*, *AvrLmS*, *LepR1*, *LepR2*, and *LepR3* were detected.

### Diversity and Evenness of the *L. maculans* Population in Manitoba

The Simpson index of diversity (IOD) was calculated to be 0.85, where an index of 1 is a random or diverse population and an index of 0 is one race (Simpson, 1949). The Simpson’s index of diversity indicated that the *L. maculans* population appears genetically diverse (Table 5). The Simpson index of evenness (IOE) was calculated to be 0.02, indicating low evenness in the population. The low evenness in the population is likely due to four dominant races that make up 61% of the population. The IOE remained low between the fall- and spring-collected samples for both years. The IOE did not change significantly between the years with an index of 0.03 in 2018 and an index of 0.04 in 2019.

**TABLE 5:** Simpson’s index of diversity (IOD) and evenness (IOE) for 254 *Leptosphaeria maculans* isolates collected from commercial canola fields in Manitoba in 2018 and 2019.

	Year		Years
Index	2018	2019	Combined
No. of races	26	20	35
IOD	0.836	0.805	0.853
IOE	0.032	0.040	0.021

### Spring vs. Fall Isolate Collection and Deployment of R Genes

The comparison of isolates between the spring and fall helps to identify the impact of resistance gene deployment. Higher frequencies of *L. biglobosa* were isolated from spring samples. An analysis of the total population was done but does not show significant differences (Figure 5). It is in specific field examples with the combination of blackleg disease levels and major resistance gene where differences are found.

**FIGURE 6:**
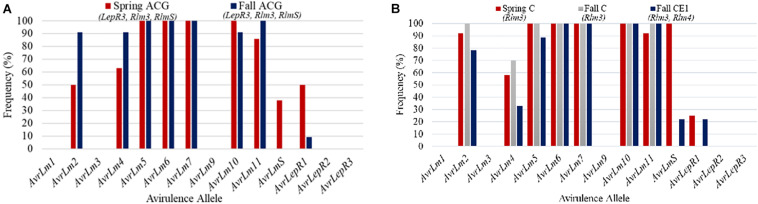
The frequency of avirulence alleles in the *Leptosphaeria maculans* population from 20 isolates collected in the spring and fall from field site MB5 in Manitoba from 2018 with a cultivar containing ACG **(A)**. The frequency of avirulence alleles in the *Leptosphaeria maculans* population from 30 isolates collected in the spring and fall from field site MB6 in Manitoba from 2018. The field site had two cultivars grown with different resistance gene groups, cultivars with C and CE_1_ **(B)**.

Field site coded as MB5 had 20 isolates collected between the spring and fall sampling in 2018. The frequency of avirulence genes for MB5 is represented in Figure 6A. Stubble collected in the spring was from a cultivar grown in 2016 with resistance gene groups ACG. The 2018 canola cultivar grown in MB5 belonged to ACG, which contains resistance genes *LepR3*, *Rlm3*, and *RlmS*. This cultivar was assessed for blackleg disease incidence and severity. The blackleg disease incidence for this field was calculated to be 64% and disease severity rating was 1.36. The major resistance genes not matching up to the *L. maculans* avirulence alleles can explain the high levels of disease incidence. *AvrLepR3* and *AvrLm3* are not found in the spring population, and only 38% of the races contained *AvrLmS* (Figure 6A). The resistance gene *RlmS* would have only been able to initiate a defense response against a small percentage of aggressive *L. maculans* races. *AvrLmS* is not recognized in the fall isolate population, suggesting a change in virulence. The *AvrLm2*, *AvrLm4*, and *AvrLm11* avirulence alleles all increased in frequency from the spring- to fall-collected isolates.

Field site MB6 from Manitoba in 2018 is an example where spring and fall isolates were compared based on the resistant cultivars grown (Figure 6B). The spring-collected isolates were from a cultivar grown in 2016 with major resistance gene group C. Two cultivars were grown in 2018 within this field and were used to assess blackleg disease levels. The difference in isolates collected from each resistance gene group (C, CE_1_) in the fall is compared to the spring isolates collected for the field (Figure 6B). The blackleg disease incidence for C was 64% and disease severity rating was 1.22, whereas CE_1_ had a disease incidence of 42% and disease severity rating of 0.76. The higher disease incidence and severity reported in C would be from *AvrLm3* in the population. The spring isolate population had a *AvrLm4* frequency of 58% (Figure 6B). This would explain why the disease incidence and severity was less in the CE_1_ cultivar. The addition of the *Rlm4* gene in resistance gene group CE_1_ would allow for the defense response in the plants to be initiated. The *AvrLm4* avirulence gene decreased in frequency between the use of C and CE_1_ by 37%; this would suggest a shift in virulence occurring where the CE_1_ cultivar was deployed.

## Discussion

The current study validated the significance of deploying different blackleg resistance gene groups in commercial canola fields in Canada’s largest canola-growing region, the prairie region of Canada, by analyzing differences in disease incidence and severity between resistance gene groups and comparing this to changes in avirulence allele frequencies. Blackleg disease incidence and severity were significantly different between resistance gene groups. The importance of knowing what blackleg major resistance gene is deployed in the canola cultivar and the frequency of avirulence genes in the *L. maculans* population helps to better steward blackleg resistance sources. The two most common *L. maculans* races in Manitoba were *AvrLm2-4-5-6-7-10-11* and *AvrLm2-5-6-7-10-11*, with 35 unique races being identified. *AvrLm6* and *AvrLm11* were found in all 254 *L. maculans* isolates collected in Manitoba (Figures 3, 5). This study provides an updated *L. maculans* race identification, frequency of races, and avirulence genes found in commercial canola fields. Knowing the blackleg major resistance gene deployed, the blackleg disease incidence and severity, along with the *L. maculans* avirulence profile causing the disease helps to measure the success of management practices and strengthen disease management recommendations.

This study only looked at one component of blackleg resistance, the major resistance genes: the other component being quantitative resistance. Low disease severity in cultivars where major resistance genes did not match the *L. maculans* avirulence frequency in the field may be explained by strong levels of quantitative resistance (Table 4 and Figure 5). To improve resistance durability, both major gene resistance and quantitative resistance must be combined to provide optimal blackleg management (Delourme et al., 2006; Brun et al., 2010). The durability and longevity of crop protection products, such as resistance cultivars, rely on using an integrated pest management approach.

At the time of this study was conducted, Canada only had four major resistance genes that were identified in commercially available cultivars. Zhang et al. (2016) reported *Rlm3* to be the most common deployed resistance gene in Canada, as it was found in over 55% of *B. napus* accessions. The high frequency of resistance gene *Rlm3* use today is most likely due from its early introduction into Canadian canola breeding programs (Gugel and Petrie, 1992). Therefore, it has been deployed in all resistant cultivars and paired with other major resistance genes. All commercially available resistance gene combinations were used in this study to provide the most relevant information to the farmer at the field level.

Both blackleg disease incidence and severity were significantly different between resistance gene groups over the 2-year study period (*P* < 0.05; Table 3). Overall, disease severity ratings were relatively low, as yield losses are not typically seen from blackleg until a disease severity rating of 2 is reached (Hwang et al., 2016; Wang et al., 2020). The study, however, did rely on natural inoculum to cause blackleg disease symptoms, so it is expected to be less than inoculated experiments. Under natural conditions, Kutcher et al. (2013) reported disease severity levels of less than 0.5. Trials that are inoculated in Canada can still experience low disease severity levels of less than 1.0 (Rashid et al., 2020). In comparison to provincial blackleg disease survey data, this study had higher disease incidence (Canadian Plant Disease Survey, 2020). This could be explained by choosing fields with high canola cropping frequency where the provincial blackleg disease survey captures fields with varying crop rotation lengths. The blackleg disease severity rating scale is subjective based on the surveyor’s perception of the level of infection. Provincial disease surveys are completed by many surveyors, whereas this project only had one individual complete the ratings. This is still noted as a potential source of error within this study.

The blackleg disease has been described as “boom and bust” in nature, because of the changes it can have in virulence (Marcroft et al., 2012). In 2003, Southern Australia experienced the breakdown of “Sylvestris” resistance, which consisted of *LepR3*, just 3 years after the commercial release of cultivars harboring it (Sprague et al., 2006). France saw increases in the frequency of virulent *avrLm1* isolates due to increased adoption of cultivars harboring *Rlm1* (Rouxel et al., 2003). These two examples, along with the Canadian *Rlm3* breakdown example, show the impact major resistance genes can have on the *L. maculans* avirulence profile (Zhang et al., 2016). Rashid et al. (2020) found a rapid loss of avirulence and shifts to virulence by *L*. *maculans* isolates in as little as 1 year in Canada. Identifying the blackleg major resistance genes within a cultivar becomes valuable to help properly steward and increase the longevity of the resistance genes (Van de Wouw and Howlett, 2019). This approach avoided yield losses of AU $13 million to Australian farmers when a warning was sent out to use different resistance genes as high disease levels were found in *LepR1* cultivars (Van de Wouw et al., 2014b). Validating the concept of strategic deployment of blackleg major resistance genes was the key objective of this study.

There are only a few labeled major resistance gene cultivars available in Canada, with some life science companies choosing not to identify the resistance genes in their cultivars. Major resistance gene *Rlm3* was the only single gene surveyed in this study, all other genes were stacked in cultivars (Table 4). Recommendations from Liban et al. (2016) not only suggested that a *Rlm6* and *Rlm7* stacked cultivar would be effective against most *L. maculans* races found in Canada but also looked at the possibility of rotating resistant genes. In Australia, rotating different blackleg resistance genes is effective in field trials (Marcroft et al., 2012). There remains knowledge gaps on how to properly rotate resistance genes and whether different resistance genes will have more impact than others (Van de Wouw et al., 2016). Stacked major gene cultivars have the potential to create races that are virulent toward several resistance genes (Rouxel et al., 2003; Sprague et al., 2006; Zhang et al., 2016; Van de Wouw et al., 2018). The discussion between the use of single resistance gene and stacked gene cultivars remains an important topic when working toward a disease management strategy.

Identifying the avirulence genes present in the *L. maculans* population in this study paves the way for a better understanding of blackleg disease pressure. The CE_1_ cultivars containing *Rlm3* and *Rlm4* were different than other resistance gene group combinations (Table 4). This is due to the addition of the *Rlm4* gene that matches up to the *AvrLm4* avirulence gene, which is frequent in the *L. maculans* population (Liban et al., 2016; Zhang et al., 2016; Fernando et al., 2018; Soomro et al., 2020). *AvrLm3* and *AvrLm9* frequencies remained low or non-existent due to the epistasis (suppression) with the presence of either *AvrLm4-7* or *AvrLm7* (Plissonneau et al., 2016; Ghanbarnia et al., 2018). With the masking of *AvrLm3*, the deployment of *Rlm3* is ineffective; this further explains the differences seen in disease incidence and severity between resistance gene groups. Zhang et al. (2016) reported a breakdown of *Rlm3* resistance, demonstrating the high evolutionary potential of *L. maculans* populations in western Canada and the overuse of the resistance gene in Canadian *B. napus* cultivars. Cultivars in this study used *Rlm3* alone or in combination with other genes, emphasizing the overuse of this gene still in Canada.

One observation from all the *L. maculans* isolate collection studies would be that the higher number of isolates collected, the higher the number of *L. maculans* races identified; however, the avirulence frequencies are remaining constant and evenness of the population low. The Simpson’s diversity index indicated that diversity was high in the overall population due to the 35 *L. maculans* races isolated (Table 5). The Simpson’s evenness index was very low, due to two dominant *L. maculans* races representing 49% of the population (Table 5). Avirulence frequency data is helpful in the development of resistance management strategies and also why there has been a heavy focus on further understanding the frequency and diversity of *L. maculans* avirulence alleles in western Canada (Kutcher et al., 2010a). The *L. maculans* population in western Canada is genetically diverse and includes avirulence alleles that are uncommon in other canola-producing regions (Dilmaghani et al., 2009). A diverse population with many avirulence alleles to match with provides options to introduce corresponding resistance genes within canola cultivars, but low evenness indicates that the focus should be on only two to three races. Identifying and knowing the predominant avirulence alleles within the population will aid in determining which resistance genes should be considered in canola breeding programs.

There has been a keen interest to develop resistant crops carrying multiple resistance conferring gene sequences through further investigation and understanding of host–pathogen interactions (Fuchs, 2017). Knowledge of gene-for-gene interactions are now being used to help farmers make informed decisions on resistant cultivars to deploy in many crops such as wheat, rice, and soybean (Gururani et al., 2012; Lof and van der Werf, 2017). The *B. napus–L. maculans* pathosystem is just one example of many, where strategic deployment of resistant sources has an impact on disease levels (Marcroft et al., 2012; Van de Wouw and Howlett, 2019). Validating the applicability of the management practice for strategic resistance gene deployment in western Canada for one pathosystem provides this as an option to manage other plant diseases in canola and other crops.

## Conclusion

Learning how to successfully deploy resistant cultivars to manage or mitigate blackleg disease in Canada is a priority not only for market access but also for the associated production losses it can cause. The validation of deployment of blackleg resistance gene groups in commercial canola fields on the Canadian prairies adds to the credibility of this management tactic, already proven to be effective in managing blackleg disease in other canola-producing regions. The applied component of this research can be incorporated into best management practices and provide farmers with information to help when choosing cultivars to effectively manage blackleg on their farms. Updated avirulence race profiles of *L. maculans* will provide plant breeders with information they need to help select resistance in their respective canola breeding programs. This information should be used as a foundation on how effective and strategic deployment of blackleg resistance genes to match *L. maculans* avirulence profile can manage blackleg disease levels in Canada.

## Data Availability Statement

The original contributions presented in the study are included in the article/Supplementary Material, further inquiries can be directed to the corresponding author/s.

## Author Contributions

JC and WF designed the experiment. JC carried out the field surveying and pathogen isolations, while ZZ, SH, and PP performed the differential tests and PCR analysis. WF, GP, and RL conceived the concept and wrote the proposal. JC completed the statistical analysis and drafted the manuscript. All authors read and approved the final manuscript.

## Conflict of Interest

The authors declare that the research was conducted in the absence of any commercial or financial relationships that could be construed as a potential conflict of interest.
