# Supplementary material for: Validating the Strategic Deployment of Blackleg Resistance Gene Groups in Commercial Canola Fields on the Canadian Prairies
Source: Front Plant Sci. 2021 Jun 10;12:669997. doi: 10.3389/fpls.2021.669997 (PMC8222824; doi:10.3389/fpls.2021.669997)
Supplement: Supplementary file 1 [file Data_Sheet_1.PDF]

Supplementary Material for Manuscript 669997

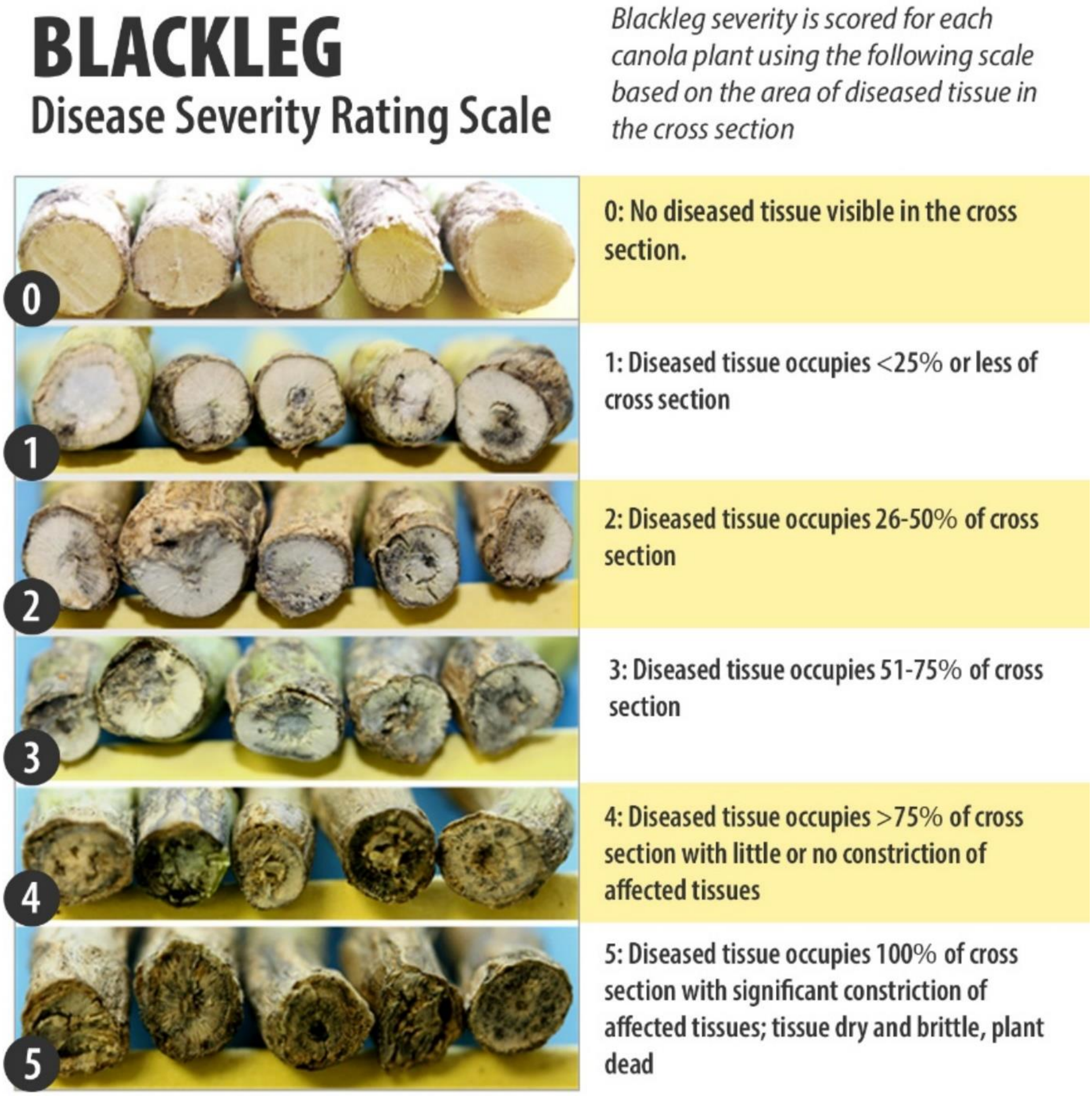

Figure S1. Blackleg disease severity rating scale based on basal cross-section infection (Canola Council of Canada, 2020).

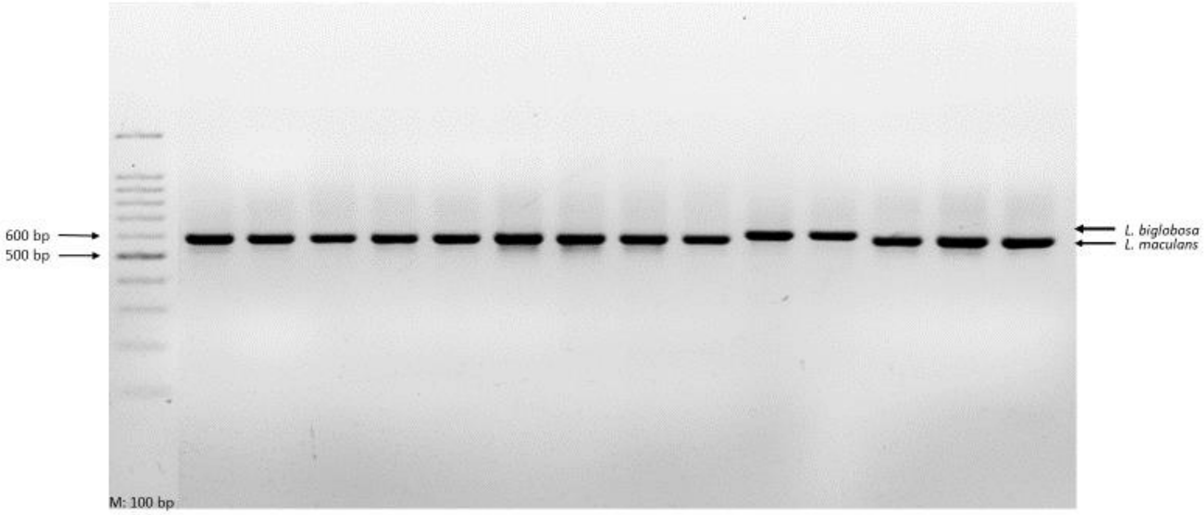

Figure S2. The primer set generates 555 to 560 bp fragment for *Leptosphaeria maculans* and a 580 to 588 bp fragment for *Leptosphaeria biglobosa* (Mendes-Pereira et al., 2003).

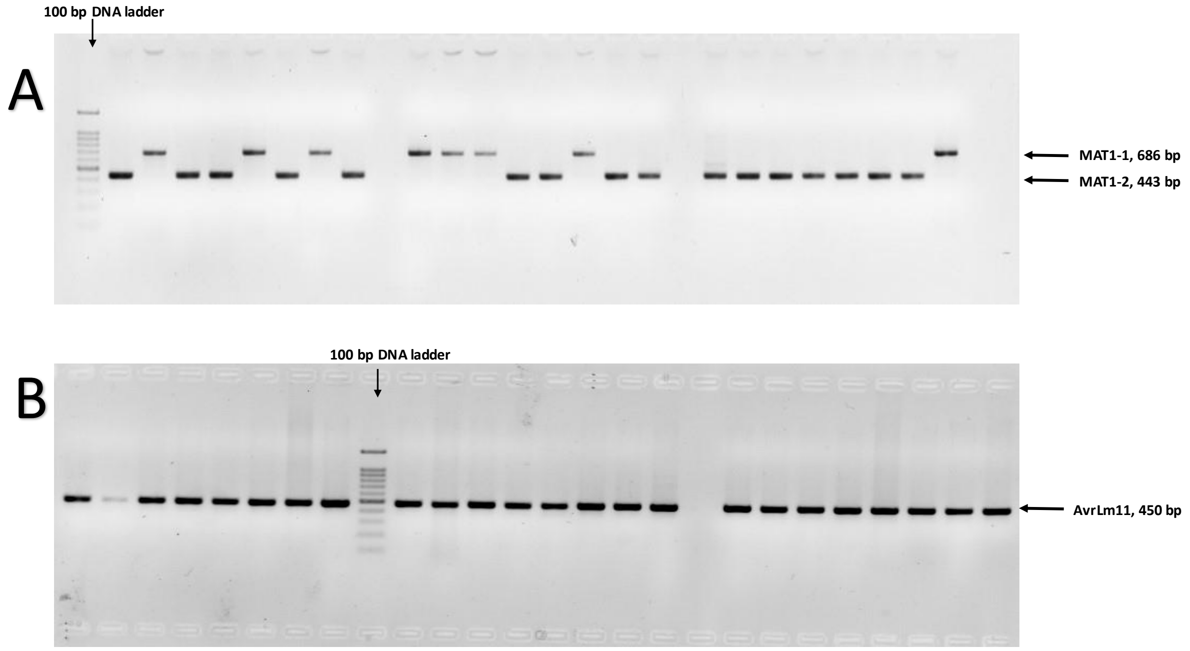

Figure S3. Photos taken from gel electrophoresis to visualize PCR results of *Leptosphaeria maculans* mating type (Cozijnsen & Howlett, 2003) (A) and of avirulence allele *AvrLm11* (Balesdent et al., 2013) (B). Photo credit: Dr. Zhongwei Zou

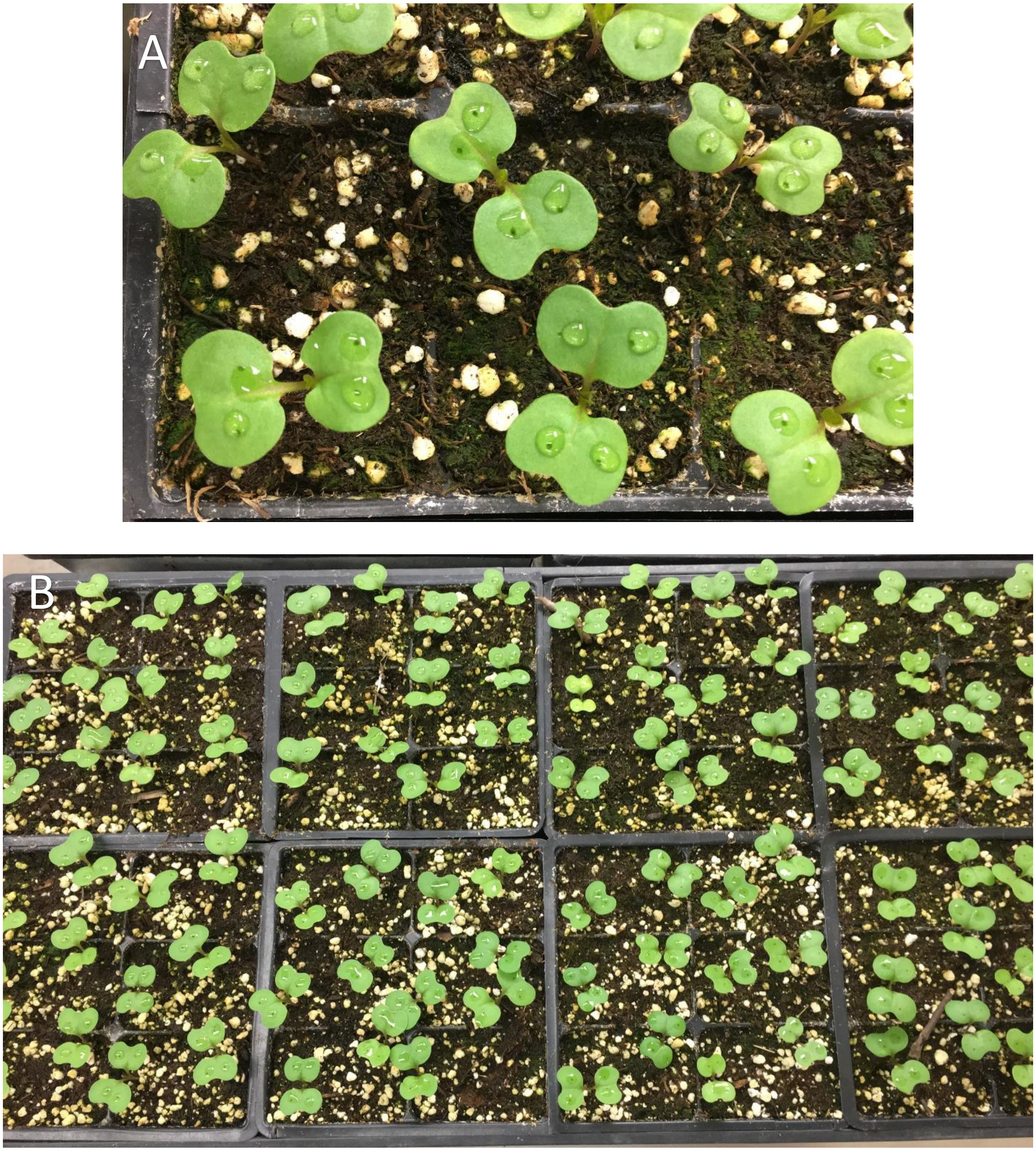

Figure S4. Seven-day old canola (*Brassica napus*) cotyledons inoculated with 10ul droplets of *Leptosphaeria maculans* pycnidiospores (A). Differential *Brassica* lines carrying known major resistance genes to observe the phenotypic reaction (B). Photo credit: Dr. Zhongwei Zou

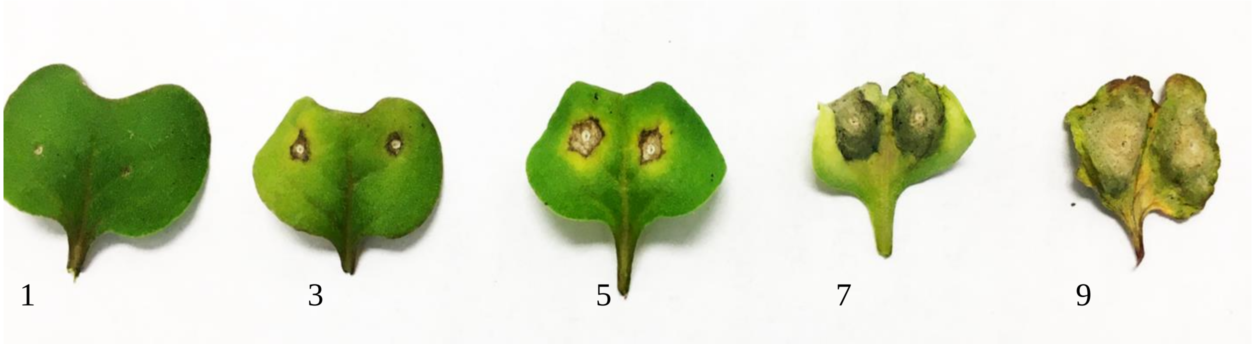

Figure S5. Symptoms on cotyledons were scored 14 days post inoculation using a disease rating scale of 0-9 (“0” indicating no infection, “9” indicating a large leaf lesion) based on lesion size, chlorosis or necrosis, and presence of pycnidia (Kutcher et al., 2007).

Table S1. Location of fields surveyed over 2018 and 2019 with their sample ID code for reference.

Year Surveyed	Sample ID	Province	Nearest Town	Legal Land Location	Previous Canola
2018	MB1	Manitoba	Newdale	NW 26-15-21-W1	2016
2018	MB2	Manitoba	Pilot Mound	NE 20-2-9-W1	2016
2018	MB3	Manitoba	Pilot Mound	SW 20-2-11-W1	2016
2018	MB4	Manitoba	Pilot Mound	NW 20-2-11-W1	2016
2018	MB5	Manitoba	Pilot Mound	SE 29-2-11-W1	2016
2018	MB6	Manitoba	Neepawa	SE 32-14-14-W1	2016
2018	MB7	Manitoba	Wayway	NW 19-20-25-W1	2016
2018	MB8	Manitoba	Wayway	S 25-20-26-W1	2016
2018	MB9	Manitoba	Inglis	SW 17-23-27-W1	2016
2018	MB10	Manitoba	Inglis	SW18-23-27-W1	2016
2018	SK1	Saskatchewan	Yorkton	SW 8-25-3-W2	2016
2018	SK2	Saskatchewan	Leroy	NW 17-35-19-W2	2016
2018	SK3	Saskatchewan	Waldheim	SW 22-42-4-W3	2016
2018	SK4	Saskatchewan	Earl Grey	NW 34-23-21-W2	2016
2018	SK5	Saskatchewan	Kennedy	NW 34-12-4-W2	2016
2018	SK6	Saskatchewan	Maymont	NE 25-4-21-W3	2016
2018	SK7	Saskatchewan	Blaine Lake	W1/2 22-42-7-W3	2016
2018	SK8	Saskatchewan	Blaine Lake	W1/2 23-42-7-W3	2016
2018	AB1	Alberta	Camrose	NE 10-46-19-W4	2016
2018	AB2	Alberta	Lacombe	NE 23-38-25-W4	2016
2018	AB3	Alberta	Carseland	NE 24-22-26-W4	2016
2018	AB4	Alberta	Berywyn	SW 26-82-24-W5	2016
2018	AB5	Alberta	Girouxville	NW 30-78-22-W5	2016
2018	AB6	Alberta	Rycroft	RL 12-79-05-W6	2016
2018	AB7	Alberta	Rycroft	NW 21-78-04-W6	2016
2018	AB8	Alberta	Turin	SW10-13-19-W4	2016
2018	AB9	Alberta	Fort Sask	NE 5-54-21-W4	2016
2018	AB10	Alberta	Fort Sask	N 8-54-21-W4	2016
2019	MB11	Manitoba	Benito	32-34-28-W1	2017
2019	MB12	Manitoba	Benito	2-34-33-28-W1	2017
2019	MB13	Manitoba	Benito	SE-23-33-29-W1	2017
2019	MB14	Manitoba	Birtle	SW-11-17-27-W1	2017
2019	MB15	Manitoba	Birtle	NW-6-17-25-W1	2017
2019	MB16	Manitoba	Birtle	NW-31-16-25-W1	2017
2019	MB17	Manitoba	Bruxcelles	NE-17-6-11-W1	2017
2019	MB18	Manitoba	Neepawa	SE-34-14-15-W1	2017
2019	MB19	Manitoba	Melita	NW-13-5-28-W1	2017
2019	MB20	Manitoba	Melita	N-15-5-28-W1	2017
2019	MB21	Manitoba	Melita	N-26-03-27-W1	2017

2019	SK11	Saskatchewan	Bruno	NE-10-37-27-W2	2017
2019	SK12	Saskatchewan	Melfort	SW-30-46-17-W2	2017
2019	SK14	Saskatchewan	Foam Lake	SE-24-28-12-W2	2017
2019	SK15	Saskatchewan	Yorkton	NW-8-25-3-W2	2017
2019	SK16	Saskatchewan	Benito	17-33-30-W1	2017
2019	SK17	Saskatchewan	Benito	32-34-30-W1	2017
2019	SK18	Saskatchewan	Kennedy	SW-27-12-4-W2	2017
2019	SK19	Saskatchewan	Gull Lake	31-12-17-W3	2017
2019	SK20	Saskatchewan	Denzil	SE2-39-25-W3	2017
2019	AB11	Alberta	Torrington	NW-8-32-26-W4	2017
2019	AB12	Alberta	Clive	SE-23-38-25-W4	2017
2019	AB13	Alberta	Joffre	SW-28-38-24-W4	2017
2019	AB14	Alberta	Joffre	SW-29-38-24-W4	2017
2019	AB15	Alberta	Consort	NE-10-35-6-W4	2017

Table S2. Type III test for fixed effects ANOVA for blackleg disease incidence and severity for all field sites comparing single resistance gene cultivars to multiple resistance gene cultivars in 2018 and 2019.

Variable	Source of Variation	df	F	Pr > F
Disease Incidence	Multiple Gene	1	1.01	0.316
	Year	1	2.15	0.146
	Multiple Gene*Year	1	1.17	0.283
Disease Severity	Multiple Gene	1	1.06	0.305
	Year	1	0.16	0.690
	Multiple Gene *Year	1	1	0.319
